# Transportation Patterns of Adults With Travel-Limiting Disabilities in Rural and Urban America

**DOI:** 10.3389/fresc.2022.877555

**Published:** 2022-04-27

**Authors:** Andrew Myers, Catherine Ipsen, Krys Standley

**Affiliations:** The Research and Training Center on Disability in Rural Communities (RTC:Rural), Rural Institute for Inclusive Communities, University of Montana, Missoula, MT, United States

**Keywords:** transportation, rural, disability, community participation, independent living

## Abstract

**Introduction:**

Lack of transportation is a significant barrier to community participation for many disabled adults. Living in a rural area introduces additional transportation barriers, such as having to travel long distances to access services or socialize, and limited public transit options. While the importance of transportation access is clear, the mix of different transportation options used by people with disabilities to participate in their communities is less understood, particularly among those who do not or cannot drive.

**Methods:**

We used data from the 2017 National Household Travel Survey to explore transportation behaviors among disabled adults in rural and urban areas and by four regions across the United States. We explored differences by transportation modalities (e.g., driver, passenger, public transportation, taxi/uber, walk) and trip purposes (e.g., social, independent living, healthcare, work). Our sample included 22,716 adults with travel-limiting disabilities.

**Results:**

Several geographic differences emerged among non-drivers. Rural non-drivers were less likely to take any trip, particularly for social activities, and reported using less public transportation or walking/rolling than urban non-drivers. Further, respondents from the Northeast were more likely to report using public transportation and walking/rolling options, relative to the Midwest, South, and West. Overall, disabled rural adults reported lower odds of giving up driving, even after controlling for socio-demographic and health characteristics.

**Discussion:**

These findings highlight the relative importance of different transportation modalities for participating in activities and the continued reliance upon personal vehicles, either as a driver or passenger, especially among rural disabled residents. Potential policy insights are discussed.

## Introduction

Transportation is important for all people, and especially people with disabilities, because it facilitates access to community participation (1–3), a key outcome of rehabilitation (4). However, compared to people without disabilities, disabled people^1^ experience limited access to transportation (5). More than 30 years since the Americans with Disabilities Act was signed into law, transportation is still a significant barrier to full inclusion in society for many people with disabilities (6). For example, ~6 million Americans with disabilities have difficulty meeting their transportation needs (7). This problem is especially salient in rural areas where transportation options are more limited (8–12).

Eliminating barriers to participating in society is one of the core themes that underpin the disability rights movement and the Americans with Disabilities Act (1990) (13–15). As such, the concept of participation has emerged as a “gold standard” for measuring outcomes in rehabilitation (4, 16). For example, the Administration for Community Living is dedicated to the vision that “all people, regardless of age and disability, […] live with dignity, make their own choices, and participate fully in society” (17).

Transportation influences the range of locations that an individual can access, and impacts the types of activities they can participate in (18, 19). More reliable and efficient transportation options can increase the range of available activities outside of the home, which is associated with increased levels of subjective wellbeing (20). Conversely, lack of transportation is a risk factor for social exclusion (21) and, in some analyses, has been found to be more impactful on how people spend their time than having a disability (22). For example, Marottoli et al. (23) found that adults over 65 who had stopped driving reported a substantial decrease in community activities even after controlling for socio-demographics and health factors. The importance of transportation for ensuring social inclusion for disabled people has been acknowledged by the United Nations Convention on the Rights of Persons with Disabilities, which identifies improvements in transportation as an opportunity to increase the social and economic participation of persons with disabilities around the world (24, 25).

In general, people with disabilities are less likely to take trips than people without disabilities; this is especially true of trips for social/recreational activities and for work (25). Disabled individuals are also less likely to travel in a personal vehicle than their non-disabled peers (26) which can restrict trip-taking behavior. For instance, Mitra and Saphores (27) found that households that do not have personal vehicles due to involuntary reasons (e.g., vehicle costs, inability to obtain insurance, or constraints due to health or age) take fewer trips. Additionally, those trips are characterized as being of shorter distance and longer duration than the trips taken by households with personal vehicles. For those who do not have access to a personal vehicle or cannot drive, including many people with travel-limiting disabilities, public transportation is instrumental to their community participation. Conversely, a lack of public transportation options can be particularly limiting.

Socioeconomic factors further exacerbate the personal and environmental factors that contribute to the experience of disability and can limit trip making. Disability prevalence is higher among people experiencing poverty (28) and poverty is increasingly connected to not owning a car or having access to a personal vehicle (29). Specifically, people with low incomes who own personal vehicles do so by taking on substantial economic burdens. Meanwhile, non-ownership of personal vehicles is increasingly associated with very low-income households. This latter association is especially true in places where the built environment is organized around personal vehicles (29), such as rural areas.

Transportation discrepancies in rural areas are exacerbated for disabled people. For instance, rural disabled people are more likely to report never using public transportation than urban disabled people (30). Among working-age disabled adults, those living in rural areas experience travel-related disabilities at slightly higher rates, but are also more likely to drive personal vehicles (31). Combined, limited public transportation services and geographically dispersed services mean that having transportation access via personal vehicle is more consequential. For example, having access to a personal vehicle in rural areas has been found to support health care utilization (8).

The United States Federal Transit Administration sponsors two separate funding mechanisms to support the transportation needs of people with disabilities and rural residents. The Enhanced Mobility of Seniors & Individuals with Disabilities program (§5310) allocates funding to states to support the transportation needs of people with disabilities and older adults (65+) where transportation is “unavailable, insufficient, or inappropriate.” For rural areas, these funds are distributed via the states' departments of transportation (32). The Formula Grants for Rural Areas program (§5311) provides funding, as well as technical assistance, to support public transportation in rural areas with populations <50,000 “where many residents often rely on public transit to reach their destinations” (33). According to an analysis of 2019 National Transit Database revenue reports (34), $327,637,963 was distributed *via* §5310 and $770,713,023 was distributed *via* §5311. However, more than half of all rural counties, containing 3.6 million people with disabilities, did not receive funds from either of these programs^2^ (35).

In summary, driving behaviors and federal funding for transportation vary significantly by geography and by disability status. While most rural Americans drive, many with disabilities cannot either because they have given up driving or because they do not have access to a vehicle. A study by Henly and Brucker (25) found that diver status was an important factor when analyzing the types of trips taken by people with and without disabilities—specifically, being a driver was associated with higher odds of taking any trip, but especially a social trip. The current paper builds upon this line of research by exploring travel patterns among rural and urban disabled adults who do not or cannot drive. We conducted an exploratory study to examine how disabled rural adults get around, relative to disabled urban adults, and how transportation use varies across regions. In particular, we examined differences between people with disabilities who were drivers and those who were non-drivers. To explore these topics, we asked the following research questions:

1. What types of trips do disabled drivers and non-drivers take in rural and urban areas?
2. What types of transportation do disabled non-drivers use and how does this vary by region?
3. What factors predict the odds of giving up driving, among adults with disabilities?

## Methods

### Data

We used data from the 2017 National Household Travel Survey [NHTS; (36)]. The NHTS is a cross-sectional survey conducted every 5–7 years to collect information about the types of trips and transportation modes used by the American public. It uses an address-based sampling frame designed to produce an equal probability sample of households, excluding group housing and institutional settings (e.g., prisons, dormitories). All respondents in a selected household complete a travel diary during a single day to document their travel behaviors. The 2017 NHTS includes 129,696 households, with 264,234 individuals and 923,572 trips. We analyzed data at the household level, individual level, and trip level to explore the travel behaviors of disabled adults in rural and urban areas throughout the U.S. The NHTS also includes weights to account for non-response and probability of being selected into the sample. More detailed information about weighting procedures can be found in the 2017 NHTS Weighting Report (37). An institutional review board approval was unnecessary because this is a secondary analysis of publicly available data.

### Measures

#### Disability Status and Assistive Devices

The NHTS asks respondents if they have “a condition or handicap that makes it difficult to travel outside of the home” (i.e., travel-limiting disability). For this paper, we consider an affirmative response to this question as someone with a disability. However, disability is not a static characteristic, and several analyses describe changes in disability status over relatively short time frames, between 4 months and 1 year (38–40), which may reflect temporary injuries. The NHTS also asks respondents if their condition has lasted for <6 months, more than 6 months, or their entire life. To focus on individuals with long-term or enduring disabilities, we excluded those with a disability lasting <6 months. The NHTS also asks if they use any of the following: cane, walker, white cane, seeing-eye dog, crutches, motorized scooter, motorized wheelchair, manual wheelchair, or other. Individuals who reported using any of these items were coded as using assistive devices.

#### Gave Up Driving

If a respondent reports a disability (or is aged over 80), the NTHS follows-up with a question asking if they have “given up driving altogether.” A “yes” response indicates that the person “has given up driving because of their disability.” We use this variable as the outcome in our logistic regression analysis. Importantly, this variable is not mutually exclusive of actually driving on their travel diary day. In a subset of cases, individuals reported “having given up driving,” but still drove. Presumably, some individuals who reported giving up driving may occasionally still need to drive, for example, if they have no alternatives or in an emergency. However, this is not clarified in any NHTS documentation.

#### Non-driver Status

The NHTS asks all respondents how many vehicles they have in their household. We defined “non-driver” status as including all individuals who reported giving up driving as well as individuals who can drive but do not have a vehicle in their household.

#### Trips

Respondents to the NHTS report every trip they take throughout their travel diary day. We used information about the main purpose for each trip to analyze the types of trips that individuals took. Each trip was assigned one purpose. “Social trips” includes recreational activities, exercise, visiting friends, and religious/community activities. “Independent Living trips” includes dropping off/picking someone up, errands, and buying meals. “Work trips” includes any trip for work or employment related activities among employed individuals. “Health trips” includes trips to the doctor's office, dentist or therapy. We excluded return trips to home and trips between different transportation modes (i.e., walking to bus stop). Types of trips were informed by another NHTS-focused study (25), however, we classified trips for healthcare purposes as a distinct category.

#### Transportation Modes

Trip records also include information about the type of transportation that was used. “Driver, personal vehicle” includes driving a car, SUV, van, truck or motorcycle. “Passenger, personal vehicle” includes riding in car, SUV, van, truck or motorcycle as a passenger. “Public transportation” includes public/commuter bus, paratransit/dial-a-ride, commuter rail, and subway/streetcar. “Taxi/rideshare” includes taxi, limo, and Uber/Lyft. “Walk/roll” includes walking and bicycle.

#### Self-Rated Health

The NHTS asks respondents to rate their general health as “excellent,” “very good,” “good,” “fair,” or “poor.”

#### Geographic Variables

The NHTS uses the U.S. Census Bureau (41) classification scheme to code households as “rural” and “urban.” Urban includes urbanized areas containing 50,000 or more people and urban clusters containing 2,500–49,999 people. Rural includes any population, housing, or territory not included in an urbanized area or urban cluster. The U.S. Census Bureau's four regional classifications are also included in these data: Northeast, South, Midwest, and West.

#### Other Characteristics

NHTS data also includes information about age, race/ethnicity, sex (male & female only), educational attainment, household income, and total number of household members. The NHTS classifies individuals as “employed” if they are 16 or older and their primary activity in the last week was either “working” or “temporarily absent from work.”

### Analyses

We merged household-level and trip-level data with person-level data, to analyze travel patterns at the individual level. We used NHTS person-level weights for all analyses. All analyses were conducted with SPSS Complex Samples Module v. 28.0. Trip data was included as both summed variables (e.g., number of trips) and as dummy variables (e.g., took any trip of that type). We used Chi-square tests to compare variables between rural and urban respondents and across regions. We used a binomial logistic regression to estimate the odds of giving up driving. Pearson correlations between variables included in the regression did not indicate multi-collinearity (42).

### Sample

Our sample includes adults (aged 18+) with a travel-limiting disability lasting for more than 6 months or their entire life (unweighted *N* = 22,716). Table 1 provides demographic characteristics on key variables for the sample of adults with a travel-limiting disability, and for the rural and urban subsamples. Rural and urban statistical differences are also reported, and showed that rural respondents were less likely to be female, to live alone, and to be formally educated, and were more likely to be White and to have at least one household vehicle than urban respondents.

**Table 1:** Weighted sample characteristics.

	**Overall**	**Rural**	**Urban**	**Sig**.
Unweighted sample	22,716	5,544 (24.4%)	17,172 (75.6%)	
Weighted sample	22,827,651	4,211,445 (18.4%)	18,616,206 (81.6%)	
Age (mean, range)	66, 18–92	66, 18–92	66, 18–92	
Age category				0.691
18–64	54.8%	55.5%	54.7%	
65 and over	45.2%	44.5%	45.3%	
Female	58.2%	51.4%	59.7%	**≤0.001**
Race/ethnicity				**≤0.001**
White (non-Hispanic)	59.5%	80.9%	54.7%	
Black (non-Hispanic)	19.3%	8.7%	21.7%	
Asian (non-Hispanic)	2.3%	0.3%	2.7%	
Multi/Other (non-Hispanic)	4.6%	4.5%	4.7%	
Hispanic (any race)	14.3%	5.6%	16.3%	
Education				**≤0.001**
High school or less	50.4%	56.4%	49.1%	
Some college	31.1%	29.7%	31.4%	
Bachelor's degree or higher	18.5%	13.9%	19.5%	
Employed	12.4%	10.4%	12.9%	0.065
Household income				**≤0.001**
< $15,000	30.2%	24.7%	31.5%	
$15,000–24,999	16.4%	20.9%	15.4%	
$25,000–34,999	13.0%	13.0%	13.1%	
$35,000–49,999	12.0%	13.0%	11.8%	
$50,000–74,999	11.5%	14.5%	10.8%	
$75,000 and higher	16.8%	13.9%	17.5%	
Self-rated health				0.094
Excellent	2.6%	2.5%	2.6%	
Very Good	9.8%	10.1%	9.8%	
Good	28.8%	24.4%	29.8%	
Fair	37.9%	40.1%	37.4%	
Poor	20.8%	22.9%	20.4%	
Uses assistive device	58.6%	59.3%	58.4%	0.662
Lives alone	25.9%	18.0%	27.6%	**≤0.001**
Gave up driving	27.8%	24.7%	28.5%	**0.036**
At least one household vehicle	80.2%	93.5%	77.1%	**≤0.001**

*P-values reported for Chi-Square tests between rural and urban columns. Bold indicates p-values < 0.05. Column totals may not sum precisely to 100% due to rounding*.

## Results

### Trips Among Drivers and Non-drivers

We were interested in understanding how access to and ability to use a personal vehicle (drivers) shaped community participation for rural and urban people with disabilities, compared to those who could not drive or did not have access to a personal vehicle (non-drivers). We analyzed all data separately for working age (18–64) and 65+ respondents to account for age-based differences in activities (e.g., retirement). Table 2 shows rural and urban analyses for drivers and non-drivers aged 18–64 and 65+. Rural and urban drivers reported similar frequencies of trips across independent living, health, social, and working domains for both working age and 65+ groups. Significant differences emerged when comparing rural and urban non-drivers, where rural working-age non-drivers reported significantly fewer trips overall, and fewer social and work trips. For those 65+ who were employed, rural non-drivers reported significantly fewer work trips.

**Table 2:** Trips taken by drivers and non-drivers with disabilities, by rural/urban (weighted).

	**Drivers, 18–64**			**Drivers, 65+**			**Non-drivers, 18–64**			**Non-drivers, 65+**		
	**Rural**	**Urban**	**Sig**.	**Rural**	**Urban**	**Sig**.	**Rural**	**Urban**	**Sig**.	**Rural**	**Urban**	**Sig**.
Any trip	63.5%	67.4%	0.229	61.2%	65.1%	0.164	46.1%	59.3%	**0.030**	34.9%	40.2%	0.286
Independent living trip	44.9%	49.6%	0.176	46.4%	50.7%	0.154	35.8%	40.8%	0.405	24.2%	27.7%	0.476
Health trip	10.8%	11.4%	0.795	8.2%	11.2%	0.062	18.7%	13.2%	0.237	9%	9.5%	0.849
Social trip	22%	24.7%	0.380	23.4%	28.2%	0.086	9.7%	22.4%	**≤0.001**	12.2%	13.3%	0.691
Work trip (employed only)	47.3%	43.6%	0.678	46.5%	38.3%	0.398	16.8%	46%	**0.026**	2.7%	28.9%	**0.008**

*P-values reported for Chi-Square tests between rural and urban columns. Bold indicates p-values < 0.05. Work trips include only respondents who were employed*.

### Transportation Modes Used by Non-drivers

Those who have given up driving or do not have access to a personal vehicle must use other means to meet their independent living, health, social, and employment transportation needs. Table 3 reports on the subset of non-driving rural and urban respondents to illustrate what transportation modes they used. Rural respondents were significantly more likely to ride as passengers in a personal vehicle, whereas urban respondents were significantly more likely to walk/roll or use public transportation. Regional differences showed that non-drivers with disabilities in the South were the least likely to use public transportation and most likely to travel as a passenger relative to other regions. This is contrasted by residents in the Northeast where over 1/3 (36%) of disabled non-drivers reported using public transportation.

**Table 3:** Transportation modes used by non-drivers with disabilities, by rural/urban and region (weighted).

	**Non-drivers, over 18**			**Non-drivers, over 18**				
	**Rural**	**Urban**	**Sig**.	**Northeast**	**Midwest**	**South**	**West**	**Sig**.
Passenger, personal vehicle	83.7%	47.8%	**≤0.001**	30.3%	54.9%	64.3%	50.2%	**≤0.001**
Walk/roll	10.1%	32.8%	**≤0.001**	50.1%	28.5%	17.6%	33.0%	**≤0.001**
Public transportation	6.2%	28.0%	**≤0.001**	36.3%	23.6%	21.5%	23.3%	**0.029**
Taxi/rideshare	1.2%	3.4%	0.093	4.8%	0.8%	3.6%	3.3%	0.200
Driver, personal vehicle	1.4%	3.1%	0.116	3.2%	6.5%	1.9%	1.0%	**0.043**
Other	5.5%	5.7%	0.964	4.4%	2.7%	5.6%	9.9%	0.087

*P-values reported for Chi-Square tests between columns. Bold indicates p-values < 0.05. In a subset of cases, some individuals reported “having given up driving,” but still drove on their travel diary day*.

### Logistic Regression on Odds of Giving Up Driving

We conducted a logistic regression to explore factors associated with giving up driving. The dependent variable was a dichotomous variable where 1 = “gave up driving due to disability.” Explanatory variables included socio-demographic, economic, health/function, and environmental variables. Socio-demographic variables included age (18–64 relative to 65+); sex (female relative to male), race (White, non-Hispanic relative to non-White), and education (some college or bachelor's degree or higher relative to high school education or less). Socio-economic variables included employment status (employed relative to not employed) and household income (< $35,000 relative to $35,000+). We measured health and function with two items. To assess health, we included indicator variables for fair, good, very good, and excellent health relative to poor health. To assess function, we included an indicator variable for using an assistive device. Finally, we included environmental variables including living alone relative to living with others, living in a rural location relative to an urban location, and living in the Northeast, Midwest, or West, relative to the South.

We hypothesized that younger age, employment, and living alone would be associated with lower odds of giving up driving due to higher need for reliable transportation. We also expected rural status would be associated with lower odds of giving up driving because there are fewer public transportation options and distances to services may preclude other transportation alternatives. We hypothesized that better health would also be associated with lower odds of giving up driving, because it may indicate less complex health issues. Similarly, we hypothesized that using an assistive device would be associated with higher odds of giving up driving because it may indicate more complex travel-limiting disabilities. We anticipated that living in the Northeast would be associated with higher odds of giving up driving, due to more transportation options. We controlled for several other sociodemographic factors, such as sex, race, education, and household income, but did not have firm hypotheses about how they would influence giving up driving.

Table 4 reports results from our logistic regression analysis. In general, model variables aligned with our stated hypotheses. However, living alone and variables to control for geographic region were not significant predictors. Having some college or bachelor's degree or higher was associated with lower odds of giving up driving, relative to those with high school education or lower.

**Table 4:** Logistic regression on odds of giving up driving (weighted).

	**OR**	**95% CI**	**Sig**.
**Socio-demographic**			
Aged 18–64 (ref: aged 65+)	0.593	0.505-0.696	**≤0.001**
Female (ref: male)	1.084	0.922–1.275	0.329
White, non-Hispanic	1.084	0.909–1.293	0.367
(ref: Non-White)			
Education (ref: high school or less)			
Some college	0.644	0.534–0.777	**≤0.001**
Bachelor's degree or higher	0.659	0.534–0.812	**≤0.001**
**Socio-economic**			
Employed (ref: not employed)	0.433	0.312–0.600	**≤0.001**
HH income < $35 K (ref: household income $35 K+)	1.060	0.887–1.266	0.522
**Health and function**			
Self-rated health (ref: poor)			
Excellent	1.322	0.712–2.456	0.377
Very good	0.595	0.441–0.804	**≤0.001**
Good	0.505	0.404–0.630	**≤0.001**
Fair	0.644	0.528–0.785	**≤0.001**
Uses assistive device (ref: doesn't use)	1.733	1.465–2.050	**≤0.001**
**Environmental**			
Lives alone (ref: doesn't live alone)	0.847	0.698–1.029	0.094
Rural (ref: urban)	0.740	0.606–0.905	**0.003**
Region (ref: south)			
Northeast	1.048	0.812–1.338	0.707
Midwest	0.917	0.730–1.152	0.457
West	1.133	0.940–1.365	0.190
Intercept	36.266		**≤0.001**

*Bold indicates p-values < 0.05. Nagelkerke pseudo R^2^ = 0.112. McFadden's pseudo R^2^ = 0.069*.

We report Nakelkerke's (pseudo R^2^ = 0.112) and McFadden's (pseudo R^2^ = 0.069) model fit statistics. Pseudo R^2^ statistics are not directly comparable to R^2^ statistics, but range from 0 to 1 and provide a benchmark for evaluating alternate models (43). Pseudo R^2^ values above 0.2 are considered to indicate good model fit. Although our model did not reach this threshold, fit statistics are comparable to other transportation models focused on people with disabilities (25).

## Discussion

Prior analyses of the NHTS have explored travel behaviors among disabled and non-disabled adults (25, 26). We expanded upon this work by describing the travel patterns within this disabled population among drivers and non-drivers. Overall, we found no significant differences in trips between disabled drivers living in rural and urban areas. Differences emerged among non-drivers (aged 18–64) whereby those living in rural areas were less likely to take a trip for any reason, but especially for social activities and work, compared to their urban counterparts. Urban residents aged 65+ were also more likely than rural residents to take a trip for work. This may indicate that the dearth of public transportation options in rural areas (9, 12, 44) impacts non-drivers more than drivers, resulting in fewer trips.

Exploring transportation modes among non-drivers revealed that rural residents relied more upon riding as a passenger in a personal vehicle compared to urban residents who were much more likely to walk/roll and use public transportation. Regionally, respondents in the South relied more on riding as a passenger in personal vehicles and less on public transit. This could be explained by the fact that rural areas throughout the South are significantly less likely to receive either §5310 or §5311 funding (34). Simply put, public transportation infrastructure may be underfunded relative to the need. However, region was not a significant factor in the regression predicting odds of giving up driving, after controlling for other factors.

We also found that individuals with better health were less likely to give up driving than those in poor health, and those who used assistive devices (e.g., cane, wheelchair) were more likely to give up driving than those who did not. This is similar to findings from Han et al. (45) who found that more than 2/3 of adults in their study gave up driving due to physical and medical challenges. Clearly, health and function can impact transportation access, specifically, being able to drive. This is an important point because being able to get around independently impacts what people do. For example, Myers and Ravesloot (22) found that disabled adults who traveled independently reported more time working and less time watching television compared to disabled adults whose transportation was dependent on others (i.e., passenger in personal vehicle) or did not travel at all. For many, particularly those living in rural areas, independent transportation is primarily facilitated by driving personal vehicles. In this way, loss of driving may have a larger impact on people living in rural areas than in urban—and, as our results indicate, are less likely to give up driving. As such, more programs are necessary to provide adequate transportation service for non-drivers in rural areas (45).

Another barrier to transportation access is housing affordability. Several individuals interviewed by the Disability Mobility Initiative of Disability Rights Washington described how many of the places they can afford to live are severely underserved by public transit systems. Many of the neighborhoods where transportation is reliable and accessible are too expensive to live. This means that these individuals are faced with a difficult choice: to either live in an affordable home with limited transportation options or live in unaffordable home with better transportation options. However, financial necessities typically demand sacrificing transportation for housing (46). Indeed, Kramer (47) found that, after controlling for income and race in urban settings, public transit access decreased as home prices decreased. The association between housing and public transportation services offers future directions for this research.

### Policy Insights

Overall, these results illustrate vast differences in transportation options for disabled adults living in rural and urban areas, particularly among non-drivers. We offer some policy insights that may help to begin addressing these inequities.

Transportation funding has historically favored urban areas. For example, a 1999 report by Seekins et al. (48) showed that urban areas, representing 75% of the total U.S. population, received approximately 94% of federal transportation subsidies. This inequity persists today in how §5310 dollars are allocated. For instance, Myers and Lissau (34) reported that rural counties (i.e., micropolitan and noncore) receive approximately 5% of §5310 funds, despite the fact that these counties account for over 18% of the disabled population in the U.S. (49). We suggest that the Federal Transit Administration make expanding funding and program capacity in these rural areas a policy priority. Further, the condition that §5311 funds be allocated to rural areas “where many residents often rely on public transit to reach their destinations” seems paradoxical. Such language seems to suggest that transportation services must first exist in order to be supported by §5311. However, there are many rural residents in need of transportation in places where services are non-existent. Funding mechanisms that can not only help maintain rural transportation services, but *establish* them, would be invaluable.

Another approach may include collaborating with faith-based organizations (FBO) to provide transportation services in rural areas. In a survey of 288 rural FBOs, Seekins et al. (50) reported that ~1/3 were willing to engage in providing transportation to people with disabilities, even people who were not members of their congregation. While the larger FBOs were most likely to own accessible vehicles, many of the smaller FBOs did not. Although, this approach is not without controversy regarding the separation of church and state. Nevertheless, the ubiquity of FBOs throughout rural America represents a potential partner in building cooperative transportation systems to serve disabled non-drivers (51).

### Limitations

There are several limitations to this study. First, the NHTS only asks about disabilities that limit travel, which excludes individuals who may experience transportation barriers unrelated to having a disability, and those who have a disability but are not limited in their transportation. This is an important point because some disabled adults may not experience limitations in their travel if they have adequate supports, thus they would not be identified in these analyses. Second, the NHTS does not ask about trips that a person does not take or about difficulties experienced while traveling. Both can impact an individual's propensity to engage in community activities which may not be captured in these data. Finally, the NHTS does not ask about modifications to personal vehicles, which is critically important for understanding the supports that people with disabilities, specifically those who use assistive devices, need to drive themselves.

## Conclusion

These findings highlight inequities across transportation access (drivers vs. non-drivers) and geography (in terms of rural vs. urban and region) among people with disabilities. Few differences appear to exist among those who can drive. However, without the ability to drive, rural residents are less likely to take any trip, but especially a trip for social or recreational activities. As such, disabled individuals in rural areas are less likely to give up driving than their peers in urban areas, even when they have difficulty traveling, potentially because doing so would significantly reduce their options for community participation. Overall, these findings indicate that more work is necessary to support disabled people who cannot or do not drive, particularly in rural areas where public transportation options are limited. To address these issues, we suggest that federal transportation funding be more equitable distributed to rural areas. Additionally, partnerships with faith-based organizations may be a potential partner toward building cooperative transportation systems.

## Data Availability Statement

Publicly available datasets were analyzed in this study. This data can be found here: https://nhts.ornl.gov/.

## Author Contributions

AM led the conceptual idea for the manuscript. The data analyses and tables were completed by AM and CI. All authors contributed to the writing, critical review, edits, and approval of the submitted version.

## Funding

This work was supported by the Research and Training Center on Disability in Rural Communities (RTC:Rural) at the University of Montana Rural Institute for Inclusive Communities under a grant from the National Institute on Disability, Independent Living, and Rehabilitation Research (grant number 90RTCP0002). NIDILRR is a Center within the Administration for Community Living (ACL), Department of Health and Human Services (HHS). The research does not necessarily represent the policy of NIDILRR, ACL, or HHS and one should not assume endorsement by the federal government.

## Author Disclaimer

The contents and opinions expressed reflect those of the authors, and should not be considered an endorsement by the funding agency or the Federal Government.

## Conflict of Interest

The authors declare that the research was conducted in the absence of any commercial or financial relationships that could be construed as a potential conflict of interest.

## Publisher's Note

All claims expressed in this article are solely those of the authors and do not necessarily represent those of their affiliated organizations, or those of the publisher, the editors and the reviewers. Any product that may be evaluated in this article, or claim that may be made by its manufacturer, is not guaranteed or endorsed by the publisher.

## Footnotes

^1^We use the terms “people with disabilities” and “disabled people” interchangeably to reflect the current guidance of disability rights advocates in the field.

^2^National Transit Database reports are not comprehensive of all transportation funding. Only recipients of §5307 and §5311 funds are required to report to the National Transit Database, recipients of other funding mechanisms report optionally.

## References

[1] Altshuler AA. Transit subsidies: by whom, for whom? J Am Inst Plan. (1969) 35:84–9. 10.1080/01944366908977578

[2] Hammel J Magasi S Heinemann A Whiteneck G Bogner J Rodriguez E. What does participation mean? An insider perspective from people with disabilities. Disabil Rehabil. (2008) 30:1445–60. 10.1080/09638280701625534 18923977

[3] Handy S. The road less driven. J Am Plan Assoc. (2006) 72:274–8. 10.1080/01944360608976749

[4] Heinemann AW. Putting outcome measurement in context: a rehabilitation psychology perspective. Rehabil Psychol. (2005) 50:6–14. 10.1037/0090-5550.50.1.6

[5] McDaniels BW Harley DA Beach DT. Transportation, accessibility, and accommodation in rural communities. In: Harley DA, Ysasi N, Bishop M, Fleming A, editors. Disability and Vocational Rehabilitation in Rural Settings. Cham: Springer (2018). p. 43–57. Available online at: https://link.springer.com/chapter/10.1007/978-3-319-64786-9_3 (accessed February 13, 2018).

[6] National Council on Disability. Transportation Update: Where We've gone and What We've Learned. Washington DC: National Council on Disability (2015). Available online at: https://www.bts.gov/archive/publications/special_reports_and_issue_briefs/issue_briefs/number_03/entire

[7] U.S. Department of Transportation: Bureau of, Transportation Statistics. Transportation Difficulties Keep over Half a Million Disabled at Home. (2017). Available online at: https://www.bts.gov/archive/publications/special_reports_and_issue_briefs/issue_briefs/number_03/entire special_reports_and_issue_briefs/issue_briefs/number_ 03/pdf/entire.pdf–

[8] Arcury TA Preisser JS Gesler WM Powers JM. Access to transportation and health care utilization in a rural region. J Rural Health. (2005) 21:31–8. 10.1111/j.1748-0361.2005.tb00059.x 15667007

[9] Arnold NL Seekins T Nelson RE. A comparison of vocational rehabilitation counselors: rural and urban differences. Rehabil Counsel Bull. (1997) 41:2–14.

[10] Bernier B Seekins T. Rural transportation voucher program for people with disabilities: three case studies. J Transport Stat. (1999) 2:61–70.

[11] Burkhardt JE Nelson CA Murray G Koffman D. Toolkit for Rural Community Coordinated Transportation Services. Washington, DC: Transportation Research Board (TCRP No. 101) (2004). Available online at: https://www.nap.edu/read/13751/chapter/1 (accessed February 11, 2022).

[12] Gonzales L Stombaugh D Seekins T Kasnitz D. Accessible rural transportation: an evaluation of the traveler's cheque voucher program. Commun Dev. (2006) 37:106–15. 10.1080/15575330.2006.10383112

[13] Baird RM Rosenbaum SE. Disability: The Social, Political, and Ethical Debate. Amherst, NY: Prometheus Books. (2008) 379 p.

[14] Cardol M Jong BAD Ward CD. On autonomy and participation in rehabilitation. Disabil Rehabil. (2002) 24:970–4. 10.1080/09638280210151996 12528676

[15] Siminski P. Patterns of disability and norms of participation through the life course: empirical support for a social model of disability. Disabil Soc. (2003) 18:707–18. 10.1080/0968759032000119479

[16] Seekins T Shunkamolah W Bertsche M Cowart C Summers JA Reichard A. A systematic scoping review of measures of participation in disability and rehabilitation research: A preliminary report of findings. Disabil Health J. (2012) 5:224–32. 10.1016/j.dhjo.2012.05.002 23021732

[17] *About ACL | ACL Administration for Community Living*. Available online at: https://acl.gov/about-acl (accessed March 7, 2022).

[18] Vilhelmson B. Daily mobility and the use of time for different activities. The case of Sweden. GeoJournal. (1999) 48:177–85. 10.1023/A:1007075524340

[19] Hägerstraand T. What about people in regional science? Papers Reg Sci. (1970) 24:7–24. 10.1111/j.1435-5597.1970.tb01464.x

[20] Bergstad CJ Gamble A Hagman O Polk M Gärling T Ettema D. Influences of affect associated with routine out-of-home activities on subjective well-being. Appl Res Q Life. (2012) 7:49–62. 10.1007/s11482-011-9143-9

[21] Kenyon S Lyons G Rafferty J. Transport and social exclusion: investigating the possibility of promoting inclusion through virtual mobility. J Trans Geogr. (2002) 10:207–19. 10.1016/S0966-6923(02)00012-1

[22] Myers A Ravesloot C. Navigating time and space: how Americans with disabilities use time and transportation. Commun Dev. (2016) 47:75–90. 10.1080/15575330.2015.1111399

[23] Marottoli RA de Leon CFM null Glass TA Williams CS Cooney LM Berkman LF. Consequences of driving cessation: decreased out-of-home activity levels. J Gerontol B Psychol Sci Soc Sci. (2000) 55:S334–40. 10.1093/geronb/55.6.S334 11078110

[24] Darcy S Burke PF. On the road again: the barriers and benefits of automobility for people with disability. Transport Res Part A Policy Pract. (2018) 107:229–45. 10.1016/j.tra.2017.11.002

[25] Henly M Brucker DL. Transportation patterns demonstrate inequalities in community participation for working-age Americans with disabilities. Transport Res Part A Policy Pract. (2019) 130:93–106. 10.1016/j.tra.2019.09.042 PMC11872224 40026835

[26] Brumbaugh S. Travel patterns of American adults with disabilities. Bureau Transport Statist. (2018) 10:1–10. Available online at: https://www.bts.gov/sites/bts.dot.gov/files/docs/explore-topics-and-geography/topics/passenger-travel/222466/travel-patterns-american-adults-disabilities-11-26-19.pdf34437518

[27] Mitra SK Saphores J-DM. How do they get by without cars? An analysis of travel characteristics of carless households in California. Transportation. (2020) 47:2837–58. 10.1007/s11116-019-09994-6

[28] Okoro CA Hollis ND Cyrus AC Griffin-Blake S. Prevalence of disabilities and health care access by disability status and type among adults — United States, 2016. MMWR Morb Mortal Wkly Rep. (2018) 67:882–7. 10.15585/mmwr.mm6732a3 30114005 PMC6095650

[29] King DA Smart MJ Manville M. The poverty of the carless: toward universal auto access. J Plan Educ Res. (2019) 1–18. 10.1177/0739456X18823252

[30] Clery E Kiss Z Taylor E Gill V. Disabled People's Travel Behaviour Attitudes to Travel. Department for Transport, London, United Kngdom. (2107) 47. Available online at: https://assets.publishing.service.gov.uk/government/uploads/system/uploads/attachment_data/file/647703/disabled-peoples-travel-behaviour-and-attitudes-to-travel.pdf

[31] Myers A Ipsen C Smith L. America at a Glance: How Do Working-Age Adults With Travel-Limiting Disabilities Get Around? University of Montana Rural Institute (2020). p. 7. Available online at: https://scholarworks.umt.edu/cgi/viewcontent.cgi?article=1065&context=ruralinst_independent_living_community_participation (accessed February 14, 2022).

[32] Enhanced Mobility of Seniors & Individuals with Disabilities - Section 5310 | FTA. Available online at: https://www.transit.dot.gov/funding/grants/enhanced-mobility-seniors-individuals-disabilities-section-5310 (accessed March 7, 2022)

[33] *Formula Grants for Rural Areas - 5311 | FTA*. Available online at: https://www.transit.dot.gov/rural-formula-grants-5311 (accessed March 7, 2022).

[34] Myers A Lissau A. America at a Glance: 5310 & 5311 Transportation Funding in Rural Counties. Missoula, MT: The University of Montana Rural Institute for Inclusive Communities (2021). p. 6.

[35] *American Community Survey 5-Year Estimates 2015-2019*. *Table S1801*. U.S. Census Bureau (2020).

[36] Federal Highway Administration. 2017 National Household Travel Survey. Washington, DC.: U.S. Department of Transportation (2017). Available online at: https://nhts.ornl.gov (accessed February 14, 2022).

[37] Dai Y. Shelley Brock Roth Jill DeMatteis. 2017 NHTS Weighting Report (2017). p. 53.

[38] Myers A Ward B Wong J Ravesloot C. Health status changes with transitory disability over time. Soc Sci Med. (2020) 244:112647. 10.1016/j.socscimed.2019.112647 31689565

[39] Ward B Myers A Wong J Ravesloot C. Disability items from the current population survey (2008–2015) and permanent versus temporary disability status. Am J Public Health. (2017) 107:706–8. 10.2105/AJPH.2017.303666 28323478 PMC5388941

[40] Sage R Ward B Myers A Ravesloot C. Transitory and enduring disability among urban and rural people. J Rural Health. (2018) 35:460–70. 10.1111/jrh.12338 30566272

[41] Ratcliffe M Burd C Holder K Fields A. Defining Rural at the U.S. Census Bureau. Washington, DC.: ACSGEO-1, U.S. Census Bureau (2016).

[42] Coakes SJ. SPSS: Analysis Without Anguish Using SPSS Version 17.0 for Windows. 1st ed. Wiley (2009). 300 p.

[43] Nagelkerke NJD. A note on a general definition of the coefficient of determination. Biometrika. (1991) 78:691–2. 10.1093/biomet/78.3.691

[44] Wood J Brown J Bond M Suguri V Florida State University . Older adult transportation in rural communities: results of an agency survey. JPT. (2016) 19:154–67. 10.5038/2375-0901.19.2.9

[45] Han D Lee Y Yu J Dejno C. How does driving status affect trip patterns among older adults in suburban and rural communities? J Trans Health. (2021) 21:101052. 10.1016/j.jth.2021.101052

[46] *Disability Mobility Initiative Disability Rights Washington*. Transportation Access for Everyone: Washington State. (2021). Available online at: https://indd.adobe.com/view/dc0a72c0-2a05-4397-a17d-3aa0ecce4923 (accessed February 15, 2022).

[47] Kramer A. The unaffordable city: housing and transit in North American cities. Cities. (2018) 83:1–10. 10.1016/j.cities.2018.05.013

[48] Seekins T Spas D Hubbard M. Inequities in Rural Transportation. Missoula, MT: The University of Montana Rural Institute for Inclusive Communities (1999). p. 4.

[49] Leopold A Greiman L. America at a Glance: An Update on Rural-Urban Differences in Disability Rates. RTC: Rural, University of Montana Rural Institute (2022).

[50] Seekins T Bridges S Santa A Denis D Hartsell A. Faith-based organizations: a potential partner in rural transportation. JPT. (2008) 11:109–25. 10.5038/2375-0901.11.1.6

[51] Seekins T. Models of Rural Transportation for People with Disabilities. RTC: Rural, University of Montana Rural Institute (2007). p. 5.

